# Clinical, radiographic and histological findings of seven teeth from two California sea lions (*Zalophus californianus*) housed under professional care

**DOI:** 10.3389/fvets.2024.1335960

**Published:** 2024-02-13

**Authors:** Ana Nemec, Margherita Gracis, Tania Monreal-Pawlowsky, William Magnone, Antonella Pritelli, Cynthia Bell

**Affiliations:** ^1^Dentistry and Oral Surgery Department, Small Animal Clinic, Veterinary Faculty, University of Ljubljana, Ljubljana, Slovenia; ^2^Department of Dentistry, Oral, and Maxillofacial Surgery, Istituto Veterinario di Novara AniCura, Novara, Italy; ^3^Department of Dentistry, Oral and Maxillofacial Surgery, Clinica Veterinaria San Siro AniCura, Milan, Italy; ^4^International Zoo Veterinary Group, Keighley, United Kingdom; ^5^Parco Natura Viva - Garda Zoologicalparc, Verona, Italy; ^6^Gardaland Sea Life, Castelnuovo del Garda, Verona, Italy; ^7^Specialty Oral Pathology for Animals, LLC, Geneseo, IL, United States

**Keywords:** California sea lion, endodontic disease, pulpitis, dental pulp polyp, dental pulp necrosis

## Abstract

Seven teeth extracted from two adult California sea lions (*Zalophus californianus*) due to pulp exposure and/or to gain access to the mandibular canine teeth were histologically evaluated, and the findings were compared with clinical and radiographic findings. Three teeth were diagnosed with pulp exposure, and two of these showed no radiographic signs of endodontic disease and were histologically vital with prominent coronal pulpitis and a pulp polyp. Another tooth with pulp exposure was showing clinical and radiographic signs of endodontic disease and was histologically confirmed with pulp necrosis. A discoloured incisor tooth was showing radiographic signs of endodontic disease and was also histologically non-vital. Two clinically and radiographically healthy mandibular first premolar teeth and one second incisor tooth had no evidence of pulpitis or pulp necrosis but had pulp canal obliteration. Regular clinical and radiographic follow-up for 5 months to 3 years after the procedures confirmed uneventful healing of the extraction sites, despite initial flap’s dehiscence. Although extractions of affected teeth in California sea lions are considered the most practical and beneficial therapy, these are associated with the risks of extensive trauma and anaesthesia and the need to perform these surgical procedures on-site under variable conditions. As California sea lions can be trained to allow conscious dental radiographic re-checks, monitoring teeth with clinical signs of pulp polyp formation and without radiographic signs of endodontic disease warrant further evaluation/reconsideration from previous recommendations. Endodontic treatment of abscessed teeth in California sea lions is reportedly unsuccessful and is discouraged. However, vital pulpectomy could be an alternative treatment to extraction in teeth with pulp polyps as it was found to be highly successful in humans, but the possibility of endodontic failure and need for further treatments should be weighted in the treatment choice.

## Introduction

As per recent reports, it is estimated that there are more than 730,000 wild and exotic animals belonging to roughly 8,500 species living under human care in 238 accredited zoos and aquariums in 31 countries of the Association for Zoos and Aquariums. However, evidence-based approach to the diagnosis and treatment of oral and dental diseases in exotic animals is still lacking, mostly due to difficulties associated with providing good follow-up evaluations on wildlife species (1).

California sea lions (*Zalophus californianus*) are carnivores with the dental formula I3/2, C1/1, P4/4, M1/1 (2), having no postnatal deciduous dentition. The development of their teeth, especially of the canine teeth, is prolonged with delayed (age not yet defined) apical closure (2, 3).

A large study on skulls of wild California sea lions revealed that most (81.7%) of the adult animals are affected by attrition/abrasion, especially males, but dental fractures were generally rare (11.7%) (2). Dental abrasion is likely related to feeding habits/behaviour of the animal as no differences in the essential minerals were detected in the teeth of the California sea lions that were more or less affected by abrasion (4).

There are only a few reports available on the dental pathology and treatment approaches of California sea lions housed under professional care (3, 5–9). Dental diseases can cause oral pain and discomfort, which are observed as changes in feeding habits and play/work-related behaviour (3, 8, 9), even though abnormal behaviour has not always been observed in animals even with severe dental wear (3). Dental abrasion and fractures may lead to endodontic infections and subsequent jaw osteomyelitis (8, 9). The most commonly affected teeth are mandibular incisor, canine, and premolar teeth (3). There is also a report of periodontitis and tooth resorption in an old Australian sea lion housed under professional care (10), even though periodontitis and tooth resorption are considered uncommon, especially in young California sea lions housed in zoological settings by some (3) but not all researchers (2).

The main purpose of this report is to describe in detail the clinical, radiographic, and histologic findings of seven teeth extracted for clinical reasons from two California sea lions housed under human care with a long-term follow-up.

## Methods

### Animals included and procedures performed

**Figure 1:**
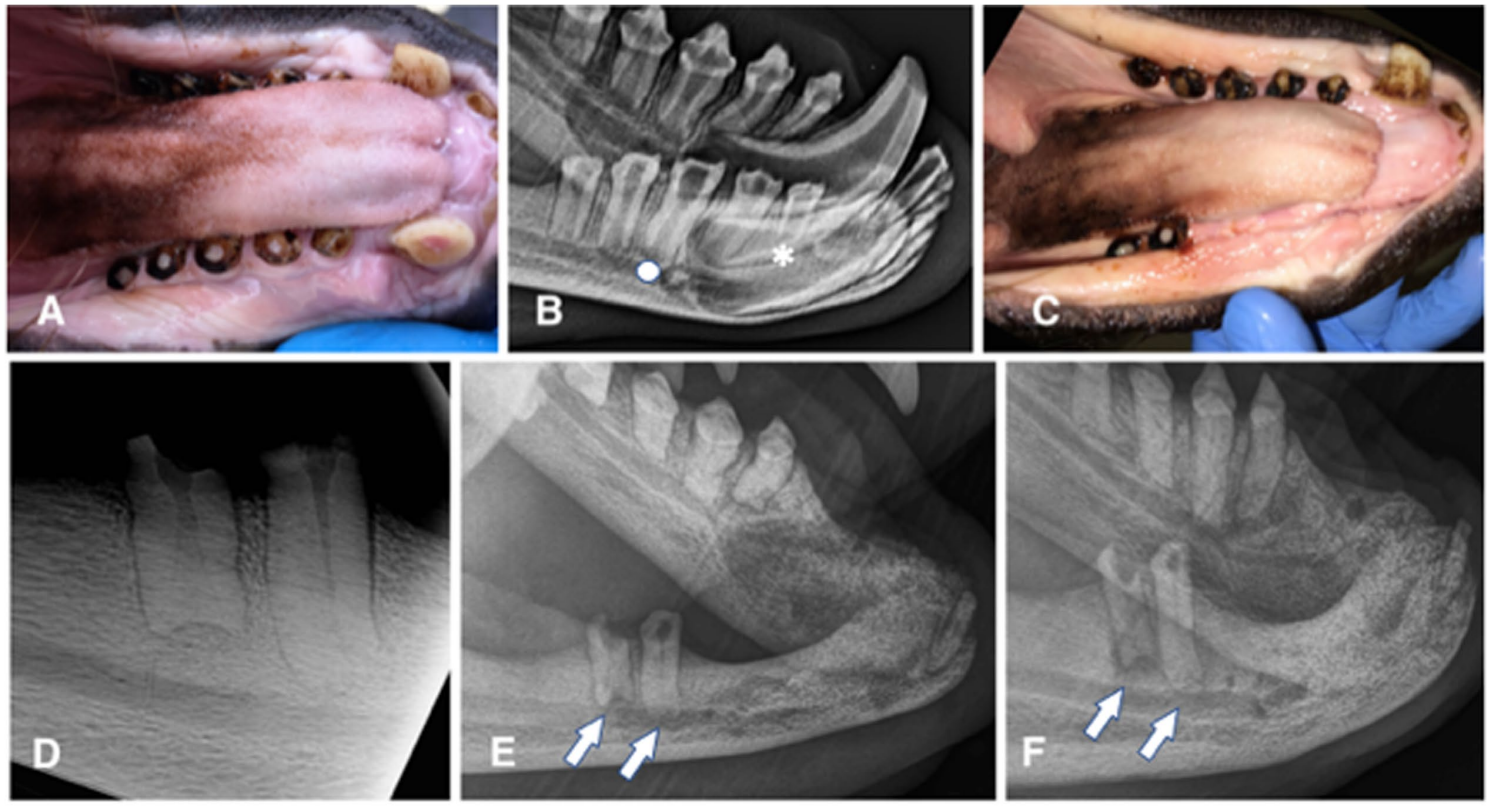
Animal #1. **(A)** clinical appearance of the right mandibular canine, premolar, and molar teeth. All teeth are abraded with pulp exposure; pulp tissue is grossly vital and protruding over the crown surface. **(B)** extraoral radiograph of the right mandible obtained 6 months later. No obvious radiographic signs of endodontic disease are noticed, except for a possibly wider pulp canal of the right mandibular first (apical asterisk) and third premolar (apical full circle) teeth. **(C)** clinical appearance of the completely healed extraction sites 6 weeks after the surgery. **(D)** intraoral dental radiograph of the remaining premolar and molar teeth of the right mandible at the time of the second surgery (44 months after radiograph from the 1B image was obtained). Despite dental pulp exposure these teeth appear to continuously develop, close the apices and remain without any obvious radiographic signs of endodontic disease. **(E)** extraoral radiograph of the right mandible obtained 10 months after the second surgery. All extraction sites are healed and there is evidence of bone formation in all previously vacated alveoli. However, at this point it appears as the remaining premolar and molar teeth developed periapical lucencies. **(F)** extraoral radiograph of the right mandible obtained 22 months after the second surgery. There is evidence of continuous bone healing of all extraction sites. Periapical disease of the remaining teeth has become even more evident, especially at the molar tooth.

Sealife Gardaland (Castelnuovo del Garda, Verona, Italy) houses three male California sea lions in an exhibit consisting of an outdoor enclosure with a pool displaying underwater theming and an indoor area with separate pools, to which the animals have access at all times. Two animals (#1 and #2) arrived from different zoological collections at 1 year of age. Both animals were in good condition on arrival, except for #1’s teeth—the animal was clinically diagnosed with pulp exposure of several teeth of the right mandible, including the canine teeth (Figure 1A). This animal developed a behaviour that consisted of biting on two areas of the underwater theming, possibly contributing further to dental abrasion. It was unknown if this behaviour was related to pain.

#### Animal #1

Animal #1 was a 2 years and 7 months old male weighing 76.6 kilograms. The animal was trained to allow for clinical photographs along with extraoral and intraoral radiographs of the mandibles to be taken (Portable 380HF, Multimage srl, Varese, Italy; SPRINT BOLT, Sound Eklin, Multimage srl, Varese, Italy) by the animal care staff and local veterinarian before the surgical procedure. These diagnostic procedures revealed abrasion of all incisor teeth, both mandibular canine teeth and all right premolar/molar teeth, with pulp exposure at the right second incisor, canine, second, third, and fourth premolar and first molar teeth. The pulp of these teeth appeared pink and slightly protruding from the occlusal surface. All dental crowns showed a dark pigmentation typical for the species (Figure 1A). The radiographic images were evaluated for signs of periodontal [i.e., widening of the periodontal ligament space and alveolar bone loss] and endodontic diseases [i.e., failure of the pulp cavity to narrow, apical widening of the periodontal ligament space, periapical lucency, and integrity of the apex/apical inflammatory root resorption] (11), as well as for other abnormalities (Figure 1B). Because of the risk for pulp infection and endodontic complications, extraction of the teeth with pulp exposure was planned. The maxillary dentition was clinically normal, and radiographs of these teeth were not performed, pending further training to allow obtaining maxillary images as well on the conscious animal.

Before the first procedure, the animal was given amoxicillin/clavulanic acid (Synulox; Zoetis Italia Srl, Roma, Italy) 22 mg/kg p.o. BID 2 days before surgery and fasted for 24 h. He was then anesthetised with 1 mg/kg zolazepam and tiletamine (Zoletil 100; Virbac Srl, Milano, Italy) and 0.025 mg/kg medetomidine (Domitor; Orion Corporation, Espoo, Finland) i.m. in the squeeze cage. After 11 min, mask induction was performed using isofluorane (Iso-Vet; 1,000 mg/g, Piramal Critical Care B.V., Voorschoten, the Netherlands) (3%) in oxygen for 5 min prior to intubation. A size 12 endotracheal tube was used for intubation, and isofluorane was kept within a range of 2–4% throughout the whole surgery with an oxygen flow rate of 3–4 L/min.

Detailed oral and periodontal examination was performed according to the American Veterinary Dental College (AVDC) guidelines for dogs and cats and adjusted for California sea lions. This confirmed findings from the awake oral exam. Intraoral dental radiographs were performed on the right mandible and rostral mandibular area using standard intraoral techniques and size 4 imaging plates (Port-X IV Portable dental x-ray, CR7 Vet Image X-ray Scanner and high-resolution reusable imaging plates; iM3 Dental Limited, Stamullen, Co. Meath, Ireland). Radiographs of the clinically healthy maxillary teeth were not performed to shorten the time under general anaesthesia.

A right mental nerve block with 1 mL of 0.5% bupivacaine (Marcaina 5 mg/mL; Aspen Pharma Trading Limited, Dublin, Ireland) and mucosal infiltration with 1 mL of 0.5% bupivacaine (Marcaina) were performed using a 2 mL hypodermic syringe with a 25G needle. An envelope flap from the right mandibular second incisor to the third premolar teeth was performed using a #15 scalpel blade. Alveolectomy over the canine tooth was performed with #6 and #8 round carbide burs on a high-speed handpiece with deionized water spray. Extraction of the right mandibular first premolar tooth to gain better access to the canine root was performed first, followed by extraction of the right mandibular canine, second incisor, and second and third premolar teeth using size 3 to 6 dental luxators and elevators (iM3, Duleek, Ireland). The canine tooth measured 47 mm in length, the second incisor tooth measured 26 mm, the first premolar tooth measured 13 mm, and the second and third premolar teeth measured 22 mm. Post-extraction alveoloplasty with a #8 diamond bur on a high-speed handpiece was performed, followed by an incision of the flap’s periosteum and slight blunt dissection to achieve tension-free closure. Before closing the wound, the area was lavaged with Ringer’s lactate and the flap was then sutured with 4–0 polyglecaprone (Monocryl; Ethicon LLC, Guaynabo, Puerto Rico, United States) in a simple interrupted suture pattern. The procedure was aborted at this point due to limited anaesthetic time decided by the young age of the sea lion and potential anaesthetic risks associated with the dive reflex in pinnipeds.

From anaesthetic injection time to the end, the procedure took 88 min. The left mandibular second incisor tooth with clinical and radiographic signs of endodontic disease, right mandibular fourth premolar, and first molar teeth were left in place because of time constraint. The animal had no access to water for 24 h. He was fed the morning after the procedure, approximately 18 h post-anaesthesia. Meloxicam (Metacam; Boehringer Ingelheim Vetmedica GmbH, Ingelheim/Rhein, Germany) 0.1 mg/kg SID and long-acting amoxicillin (Clamoxyl; Zoetis Italia Srl, Roma, Italy) 22 mg/kg BID were started i.m. during anaesthesia and then continued p.o. for 7 days.

Partial dehiscence of the flap was observed 14 days after the procedure. In the area of dehiscence, the sutures were still present, some on the flap and some on the gingiva on the lingual side of the extractions, but laceration of the tissues on the opposite side where the sutures had been placed was evident. Conservative treatment with meloxicam (Metacam) at 0.1 mg/kg p.o. SID for 5 days and amoxicillin/clavulanic acid (Synulox) at 10 mg/kg p.o. BID for 10 days, and daily lavage with 0.12% chlorhexidine solution were elected. Complete soft tissue healing by second intention was observed 1 month after the procedure (Figure 1C). The extraction sites were monitored radiographically, showing slow but proper bone healing (i.e., evidence of new bone formation in the vacated alveoli) (Figures 1D–F).

**Figure 2:**
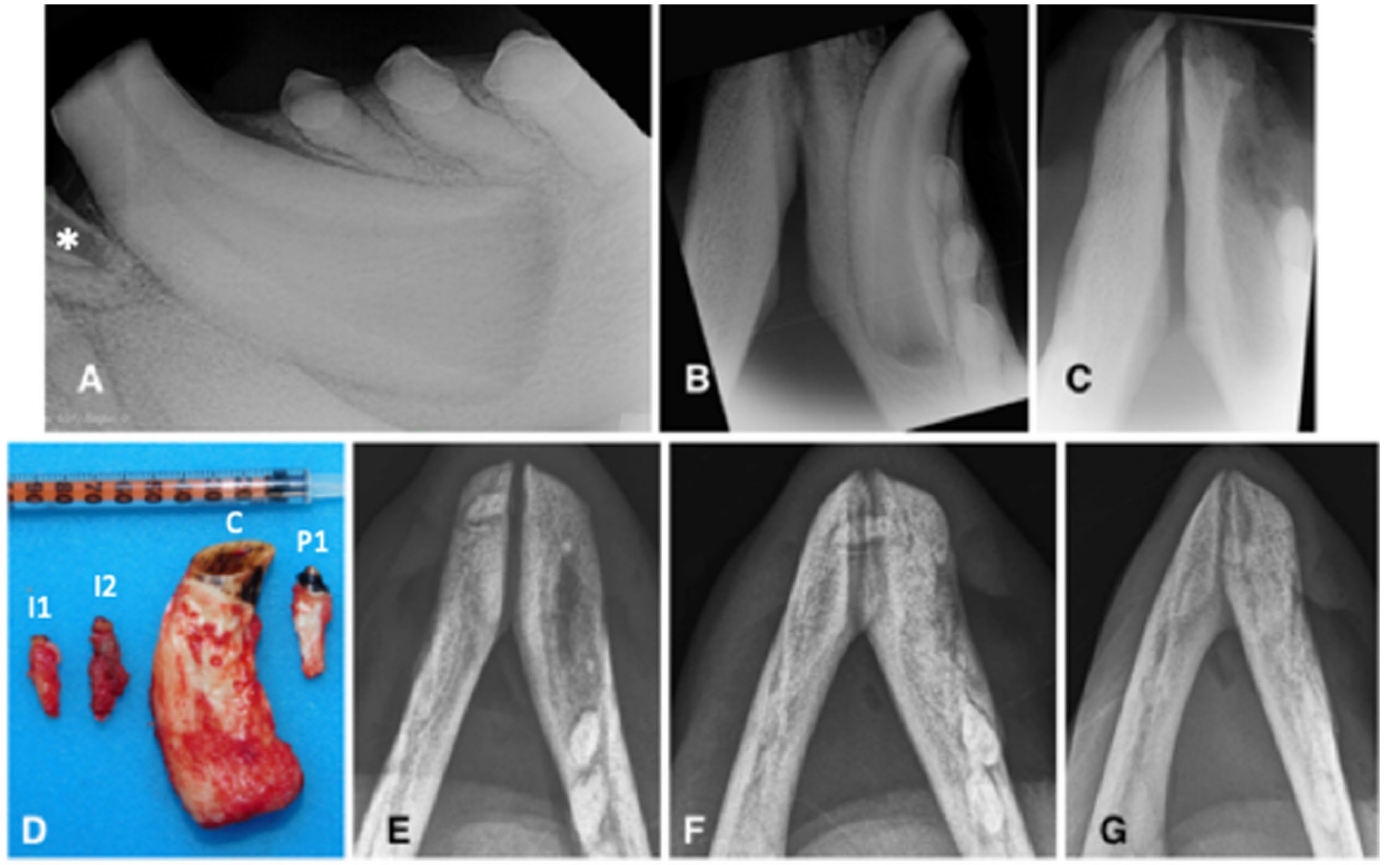
Animal #1. **(A)** preoperative lateral intraoral dental radiographic view of the left mandibular canine tooth at the time of second surgery. Although the left mandibular second incisor tooth is not entirely visualized, there is visible inflammatory resorption of the tooth apex (asterisk), indicative of endodontic disease of this clinically discolored tooth. **(B)** preoperative occlusal intraoral dental radiographic view of the left mandibular canine tooth at the time of second surgery. The extraction site of the right mandibular canine tooth has completely healed. There are no obvious radiographic signs of endodontic disease despite pulp exposure of the severely abraded canine tooth. **(C)** immediate post-operative intraoral dental radiograph of the vacated alveoli of the left mandibular incisor and canine teeth. **(D)** the extracted teeth (I1: first incisor; I2: second incisor; C: canine; P1: first premolar). **(E-G)** intraoral radiographs (occlusal view) obtained, respectively, 10, 15, and 22 months after the (second) surgery demonstrate continuous healing and remodeling of the rostral mandibles post dental extractions.

The patient was examined again 3 years later and found to have further dental abrasion and pulp exposure of the left mandibular second incisor and canine teeth. At that time, the animal was prepared and anaesthetized as described for the first procedure, and dental radiographs of the right mandible (Figure 1D) and the affected teeth on the left side (Figures 2A,B) were performed. These revealed no radiographic abnormalities of the left teeth, except for a wider pulp cavity of the discoloured left mandibular second incisor tooth as compared with the contralateral tooth, suggestive of failure of the pulp cavity to narrow due to non-vitality of the tooth. The previous extraction sites looked clinically and radiographically completely healed (Figure 2B).

Left mental nerve block and mucosal infiltration at the left canine and incisive area were performed with 2 mL of 2% lidocaine (Lidocaina 2%; Ecuphar Italia Srl, Milano, Italy). Initially, a full-thickness triangular mucogingival flap was created with a vertical incision at the mesiobuccal line angle of the left canine tooth. The alveolar incision was extended to the left second premolar tooth and then to the first incisor tooth. The left mandibular first and second incisor teeth, canine tooth, and first premolar tooth were surgically extracted (Figure 2C) and submitted for histopathological analysis. The canine tooth measured 57 mm in length, the first incisor tooth measured 17 mm, and the second incisor and first premolar teeth measured 23 mm (Figure 2D). The flap was sutured as described before. The right mandibular fourth premolar and first molar teeth were left in place, showing no radiographic signs of endodontic disease and possibly normal development, despite long-term pulp exposure (Figure 1D).

The animal was kept out of the pool for 24 h and then allowed to get back into water. He was fed the morning after the procedure, approximately 15 h post anaesthesia.

Partial dehiscence of the flaps was observed 2 days after the procedure and was similar in appearance after the first procedure. Conservative management was again elected and resulted in complete healing by second intention. Bone healing was monitored radiographically with intraoral and extraoral techniques at 6, 8, 10, 15, 19, and 22 months after the procedure, showing again slow but proper healing of the extraction sites (Figures 2E–G). At the 10 months radiographic re-examination from the second procedure, the right mandibular premolar teeth started showing signs of endodontic disease (Figure 1E).

#### Animal #2

**Figure 3:**
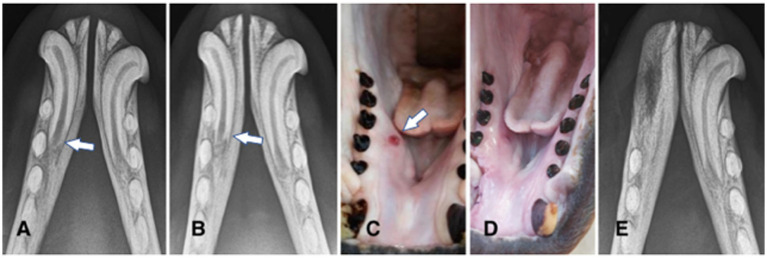
Animal #2. **(A,B)** intraoral radiographs (occlusal view) obtained 6 months apart reveal severe inflammatory root resorption and periapical lesion of the right mandibular canine tooth (arrows). **(C)** clinically visible draining tract (arrow) in the area of the right mandibular canine tooth apex corresponds with the radiographic findings on the intraoral radiographs. **(D)** the draining tract disappeared and the extraction site was completely healed 5 weeks after the surgery. **(E)** intraoral radiograph (occlusal view) obtained 8 months after the surgery demonstrates healing post extraction of the right mandibular incisor, canine and premolar teeth.

Animal #2 was a 7-year-old male weighing 166 kilograms. The animal was trained to allow for certain diagnostic procedures while conscious. Clinical photographs along with intraoral and extraoral radiographs were obtained by the trainers and the local veterinarian before the surgical procedure and revealed abrasion, pulp exposure, and endodontic disease affecting the right mandibular canine tooth, with a draining tract opening on the lingual side of the mandible, at the level of right mandibular second and third premolar teeth (Figures 3A–C). Maxillary dentition was clinically normal, therefore dental radiographs were pending, further training to allow obtaining additional radiographs on a conscious animal. The animal had been treated with intermittent courses of antibiotics of 1 week duration each [amoxicillin-clavulanic acid (Synulox) 10 mg/kg p.o. BID and enrofloxacin (Baytril; Bayer Animal Health GmbH, Leverkusen, Germany) 5 mg/kg p.o. BID] a year before due to swelling of the right mandible, and a small draining tract on the lingual side, apical to the third premolar tooth. Abrasion was also affecting all mandibular incisor teeth.

The animal was premedicated with 0.9 mg/kg zolazepam and tiletamine (Zoletil 50/50, Virbac Srl, Milano, Italy) and 0.02 mg/kg medetomidine (Domitor) i.m. Within 5 min, an initial effect could be observed, and the animal positioned itself in sternal recumbency 10 min after initial injection, but eye movement could still be detected. When being approached, the animal showed bouts of hyperexcitability. An attempt to mask the animal with isofluorane (Iso-Vet) was done, where the animal suffered again from another bout, and when a roll up movement of both eyes was observed and no effect of the isofluorane detected, the procedure was aborted. The animal was reversed with atipamezole (Antisedan; Orion Corporation, Espoo, Finland) at 0.1 mg/kg i.m. 48 min after the initial injection and within 2 min he was fully awake.

After 6 months, the animal was planned for surgery again. Three days before the surgery, the animal was started on amoxicillin/clavulanic acid (Synulox) 10 mg/kg p.o. BID and a day before surgery on meloxicam (Metacam) 0.1 mg/kg p.o. SID. He was premedicated with 1 mg/kg zolazepam and tiletamine (Zoletil 50/50) and 0.02 mg/kg medetomidine (Domitor) i.m. Within 3 min, an initial effect could be observed, and the animal was positioned in sternal recumbency 7 min after initial injection, but eye movement could still be detected. After 15 min, further medetomidine (Domitor) was given i.m., to reach 0.025 mg/kg and a desired effect. After 3 min, mask induction was performed using isofluorane (3%) (Iso-Vet) in oxygen for 25 min prior to intubation. A size 16 endotracheal tube was used for intubation, and isofluorane was kept within a range of 3–5% throughout the whole operation with an oxygen flow rate of 3 to 4 L/min. Ringer’s lactate (Ringer Lattato SALF S.p.A., Laboratorio Farmacologico, Bergamo, Italy) and saline (Socio Cloruro 0.9%; B Braun Milano S.p.A., Milano, Italy) solutions were administered s.q., meloxicam (Metacam) at 0.1 mg/kg SID and amoxicillin/clavulanic acid (Synulox) at 10 mg/kg BID were injected i.m. and continued orally for 1 and 2 weeks after the surgery, respectively.

Detailed oral examination and dental radiographs were performed as described for animal #1 and confirmed findings from awake oral exam. Like in animal #1, the maxillary dentition was clinically normal, and radiographs of these teeth were not performed.

Due to inability to locate the middle mental foramen for an effective nerve block and provide at least desensitization to the soft tissues, infiltration anaesthesia with 2 mL of 2% lidocaine (Lidocaina 2%) was performed using a 2 mL syringe with a 25G needle. Surgical extraction of the right mandibular canine tooth was achieved via a full-thickness triangular flap from the right mandibular second incisor tooth to the second premolar tooth, extraction of the second incisor and first premolar teeth to improve access to the canine’s root, buccal alveolectomy, tooth luxation and elevation, alveoloplasty and flap suturing without tension with 4–0 polyglecaprone (Monocryl), as described for animal #1. Before flap closure, a splash block in the alveoli of the extracted teeth with 2 mL of 2% lidocaine (Lidocaina 2%) was performed. Extracted teeth were submitted for histopathological analysis.

After 1 h and 33 min of initial injection, the animal was reversed with 0.125 mg/kg atipamezole (Antisedan) i.m., was extubated after 20 min, and fully awake in 1 h. The animal was not allowed in the water overnight and was fed the next morning.

Partial dehiscence of the flap was noticed 2 days after the procedure and was managed conservatively as before in animal #1. The draining tract healed in 2 weeks, and complete soft tissue healing of the extraction wound was achieved in 5 weeks (Figure 3D). Intraoral radiographs were obtained on follow-ups at 4, 8, and 11 months and revealed slow but uneventful healing of the extraction sites (Figure 3E).

### Preparation of the extracted teeth for histopathological examination

The extracted teeth were immediately placed in 10% buffered formalin, and 7 teeth were submitted for histopathological analysis. After fixation and decalcification with hydrochloric acid, the teeth were processed routinely, sectioned, and stained with hematoxylin and eosin (H&E).

## Results

### Histological findings of the extracted teeth

#### Animal #1

**Figure 4:**
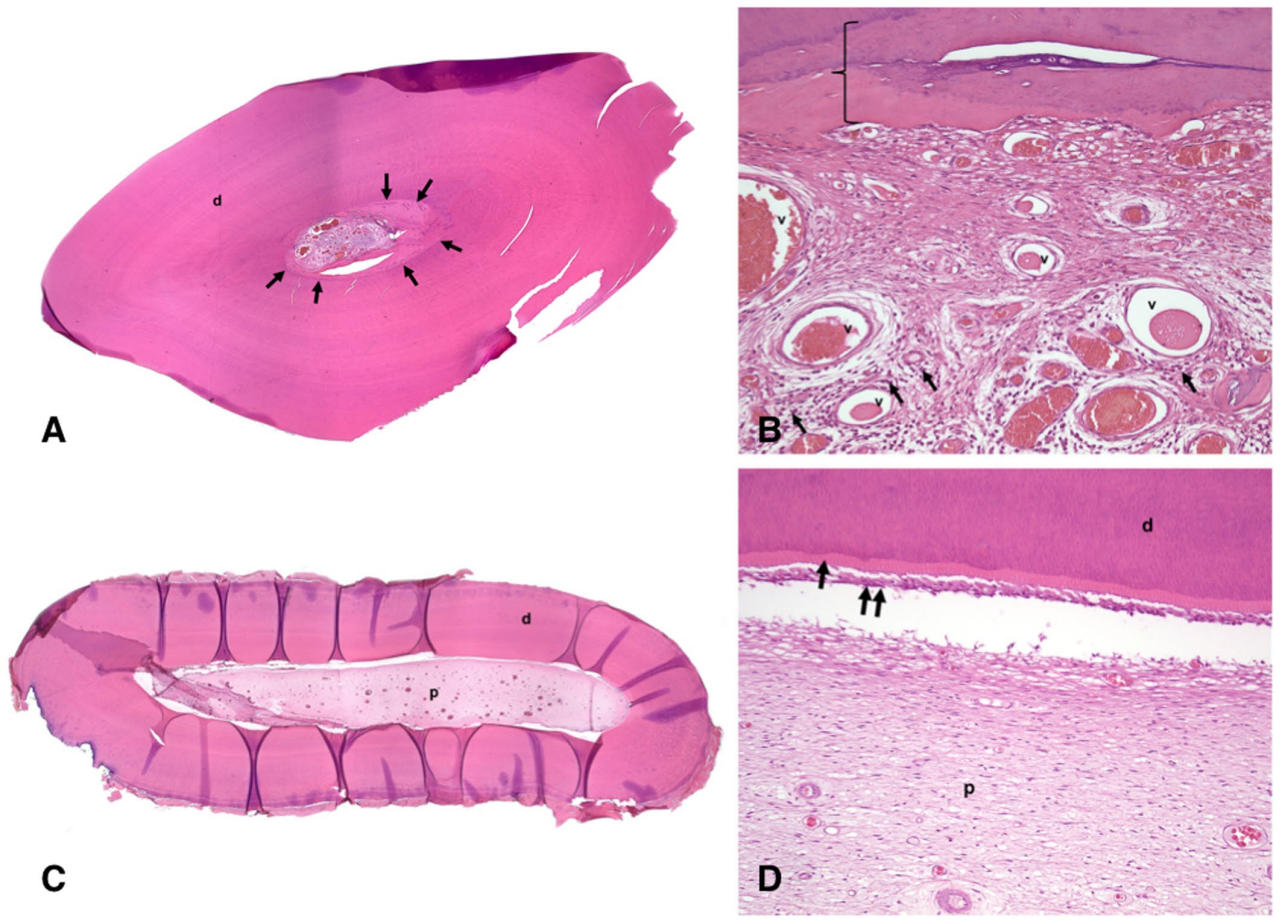
Histological images of the left mandibular canine tooth of the animal #1; H&E stain. **(A)** low magnification histological image of a section through the crown of the decalcified tooth has uniform dentin (d) except immediately surrounding the pulp chamber (arrows). **(B)** high magnification histological image of the section in Figure 4A. The vital pulp stroma with many congested blood vessels (v) and moderate numbers of inflammatory cells (small dark foci indicated by arrows). The innermost layer of tertiary dentin resembles bone (bracket). **(C)** low magnification histological image of a section through the root of the decalcified tooth has normal dentin (d) and uniform pulp stroma in the pulp chamber (p). **(D)** high magnification histological image of the section in Figure 4C. There is normal organization of dentin (d), predentin layer (arrow), odontoblast layer (double arrow), and pulp stroma (p) without inflammation.

Histopathological evaluation of extracted teeth revealed one tooth with pulp necrosis (left mandibular second incisor tooth), two vital teeth with chronic hyperplastic pulpitis (left mandibular first incisor and canine teeth), and one vital tooth without pulpitis or pulp necrosis (left mandibular first premolar tooth). The non-vital left mandibular second incisor tooth had a wide pulp chamber due to pulp necrosis and premature cessation of dentinogenesis. The pulp chamber contained neutrophils, macrophages, and necrotic debris with bacteria. Similar inflammation was present in the periapical fibrovascular tissue and bone. Chronic hyperplastic pulpitis in the left mandibular first incisor and canine teeth was most severe in the crown while inflammation diminished and was absent or minimal within pulp at the root apex (Figure 4). The canine tooth had particularly exuberant and inflamed granulation tissue at the occlusal surface of the crown, corresponding with the pink pulp tissue that was observed to have protruded from the tooth. Each of the vital teeth had narrowing of the pulp canal due to over-production of dentin, including both tubular secondary dentin and bone-like tertiary dentin, which is a characteristic of pulp canal obliteration (Figures 4A,B). In each of these teeth, over-production of dentin was most pronounced in the crown.

#### Animal #2

Histopathological evaluation of extracted teeth revealed a non-vital right mandibular canine tooth and two teeth (right mandibular second incisor and first premolar teeth) with pulp canal obliteration. The canine tooth had abundant necrotic debris within the pulp chamber including numerous coccobacilli bacteria. Although the incisor and the premolar were presumed to have been vital, the histological sections did not clearly demonstrate vital pulp as the pulp chambers were partially obliterated by dentin.

All data comparing major clinical, radiographic, and histological findings are presented in Supplementary Table S1.

## Discussion

To the authors’ knowledge, this is the first report that describes in detail the clinical, radiographic, and histologic findings of teeth in California sea lions (*Zalophus californianus*) with a long-term follow-up.

The most common lesions in the skulls of wild California sea lions and Steller sea lions *(Eumetopias jubatus)* were attrition/abrasion and periodontitis, with males and adults more commonly affected. This population also suffered more commonly from dental fractures, although this pathology was relatively rare in the wild animals (2, 12). On the contrary, both animals in this report had complete adult dentition without clinical and radiographic evidence of periodontal disease. However, similarly, many mandibular teeth were affected with dental wear (3, 7). In wild California sea lions, wear pattern of the canine teeth has been reported mostly on the mesial and distal aspect of the teeth (2), while in these animals, severe abrasion leading to dental pulp exposure was on the buccal (animal #1) and mesial side (animal #2) of the canine crowns. The premolar teeth of animal #1 were reduced to a flat surface as reported before for California sea lions housed under professional care (3). Likely, the pattern and degree of wear are a sequel of feeding habits and an abnormal behaviour. Indeed, animal #1 was observed chewing on the pool walls. This behaviour has not been observed again since the surgery was performed, possibly indicating oral discomfort before the dental treatment.

In dogs and cats, pulp exposure, if left untreated, inevitably leads to pulpitis, pulp necrosis, and apical periodontitis (13, 14). Periapical lesions were not commonly observed on the skulls of wild California sea lions, but the data may underestimate the problem due to missing imaging in that study (2). Endodontic disease evident as radiographically visible periapical lesion and/or clinically visible draining tract was observed in some teeth of the sea lions included in this report and previous reports (3, 7). Moreover, in this report, both teeth with or without pulp exposure that were showing clinical (i.e., discolouration, draining tract) and radiographic (i.e., failure of the pulp cavity to narrow, periapical lucency, and apical root resorption) signs of endodontic disease were also histologically confirmed to have pulp necrosis. On the other hand, both teeth with pulp exposure yet without other obvious clinical and radiographic signs of endodontic disease were histologically diagnosed as vital with coronal hyperplastic pulpitis. These two teeth also showed thickened dentin wall with narrowing of the pulp canal, but this may not necessarily be related to pulp exposure and disease since pulp canal obliteration was noted in clinically healthy teeth (i.e., those extracted to gain access to the canine teeth). Although histological examination is missing, long-term continuous vitality of the teeth despite pulp exposure can also be presumed for the right premolar and molar teeth that were not extracted due to time constraints, as they showed no radiographic signs of endodontic disease (and likely normal development) over a period of 3 years. However, at that point, radiographic signs of endodontic disease appeared obvious.

Notably, the formation of “pulp polyps” associated with pulp vitality has been reported in (young) California sea lions (housed under professional care) with large pulp cavities, despite pulp exposure (3, 7). Chronic hyperplastic pulpitis lesions (pulp polyps) are rare in humans but occur more commonly in teeth of children and young adults than in adults (15, 16). Young teeth, especially with open apices allowing for an excellent blood supply, that develop pulp polyps may resist bacterial infection and necrosis (16).

Open extraction of canine teeth and closed extraction of premolar/molar teeth in California sea lions housed under professional care were described before and are considered the most practical and beneficial therapy in the case of endodontic disease (3, 6–9). The extraction of mandibular incisor and premolar teeth in the animals described here was subjectively considered easy, as the size of the roots is small and the shape is cylindrical and straight or only slightly angled. However, the mandibular canine teeth are curved distally and have a very pronounced trapezoid shape with a wide apex as compared with the coronal portion of the root. For this reason, extensive alveolectomy is necessary to complete the dental extraction of the canine teeth and to minimize the chances for root fracture. In fact, extraction of the ipsilateral first premolar and second incisor teeth was performed to achieve better access to the canine root and to be able to extend the ostectomy to the canine distal root surface.

Dehiscence was possibly due to soft tissue contracture and the lack of appropriate flap’s bony support, as a large osseous defect was left after the extraction. However, in the absence of tissue tension upon closure of the extraction sites, it is more likely that some type of trauma occurred to the flaps. This hypothesis may be supported by the fact that the sutures were not lost but still in place. In fact, dehiscence seems to be a common complication after open dental extractions in California sea lions, but wounds usually heal completely by second intention without further surgical intervention (3, 9), as also observed in our cases.

Due to reported (and also observed in our cases) traumatic/difficult extraction of the canine teeth, high surgical extraction site dehiscence rate, and inability to assure aseptic approach in a suboptimal working environment and potentially devastating severe complications to extractions (e.g., osteomyelitis), antibiotics were administered during the perioperative period in both animals. This is not the standard of care in veterinary dentistry for dogs and cats without systemic signs of disease (17). For concerns of severe morbidity, animal #2 was also treated with antibiotics at the time of mandibular swelling, as it was impossible to immediately surgically address the endodontic disease. In view of antibiotic overuse, such approach will need to change for veterinary dentistry, once we grow our knowledge of the disease processes, treatments, and general approaches.

Any tooth with clinical and/or radiographic signs of non-vitality should be treated as soon as possible to avoid persistent and/or spreading endodontic infection. As the extraction of canine teeth in sea lions is a relatively traumatic and potentially long procedure, with correlated anaesthetic risks (cardiac arrest, fatal apnoea due to the diving reflex, hypothermia and hyperthermia, prolonged recoveries, and death) (18), alternatives to dental extractions should also be considered. The fact that these animals can be trained to allow conscious dental radiographic re-checks (5, 7, 10), as opposite to dogs and cats (19, 20), monitoring of teeth with clinical signs of pulp polyp formation and without radiographic signs of endodontic disease warrants further evaluation/reconsideration from previous recommendations (3, 6, 8). Although hyperplastic pulp usually has no symptoms in human children (discomfort may be observed during mastication if moveable tooth fragments are present) and treatment may be delayed for up to a year after dental trauma (16), it should be highlighted that the level of pain caused by pulp exposure may be difficult to evaluate objectively in animals. In addition, it is likely that teeth with pulp polyps become non-vital over time, as suggested by the radiographic monitoring of the right mandibular premolar and molar teeth of animal #1. Therefore, teeth with chronic hyperplastic pulpitis still need to be treated, but the treatment may be delayed and treatment options explored.

Although endodontic treatment of abscessed teeth in California sea lions is reportedly unsuccessful and is discouraged (6), it has to be noted that endodontic treatments are successful and less traumatic alternatives to large teeth extraction in dogs and cats. Failure of endodontic treatment may be related to the pre-existing condition of the affected tooth, (inappropriate) treatment choice, and/or technique and materials used (21–28). Therefore, potential for endodontic treatment of the teeth with exposed pulps in sea lions should be reconsidered and elaborated based on the presenting clinical scenario (e.g., vital vs. non-vital teeth, closed vs. open apex of the tooth), to choose the most appropriate endodontic treatment type. Vital pulpectomy as performed in dogs (23, 29) with proper radiographic monitoring of the treated tooth/teeth could be a valid alternative treatment to extraction in sea lions, particularly for immature permanent canine teeth, possibly even in the case of chronic hyperplastic pulpitis. In fact, vital pulpectomy was found to be highly successful in humans (30), especially as the radicular pulpal tissue in teeth with pulp polyps was shown to remain normal in longer periods after pulp exposure (31) and also observed in teeth in this report. Other endodontic treatment options (i.e., direct pulp capping, regenerative endodontics, and apexification), especially for immature permanent teeth, should also be considered (32). However, the possibility of endodontic failure and need for further treatments should be weighted in the treatment choice.

In conclusion, dental radiographs should be a routine part of oro-dental examination in sea lions and can be obtained on conscious animals if properly trained. Any fractured tooth with exposed pulp should be treated, but timing of the treatment and treatment choice need to be based on the combination of clinical and radiographic signs. Vital pulpectomy with proper radiographic monitoring of the treated tooth/teeth could be a valid alternative treatment to extraction, particularly for immature permanent canine teeth even with chronic hyperplastic pulpitis.

## Data availability statement

The original contributions presented in the study are included in the article/Supplementary material, further inquiries can be directed to the corresponding author.

## Ethics statement

Ethical approval was not required for the studies involving animals in accordance with the local legislation and institutional requirements because these animals were patients and treated as clinically indicated. The letter addressing ethical concerns and client agreement is uploaded. Written informed consent was obtained from the owners for the participation of their animals in this study.

## Author contributions

AN: Conceptualization, Data curation, Investigation, Methodology, Project administration, Writing – original draft. MG: Data curation, Investigation, Methodology, Resources, Writing – original draft. TM-P: Writing – original draft. WM: Writing – review & editing. AP: Writing – review & editing. CB: Conceptualization, Data curation, Formal analysis, Investigation, Methodology, Writing – original draft.
