# Supplementary material for: Clinical, radiographic and histological findings of seven teeth from two California sea lions (Zalophus californianus) housed under professional care
Source: Front Vet Sci. 2024 Feb 13;11:1335960. doi: 10.3389/fvets.2024.1335960 (PMC10896890; doi:10.3389/fvets.2024.1335960)
Supplement: Supplementary file 1 [file Table_1.DOCX]

| Animal | Tooth | Pulp exposure | Other clinical findings | Radiographic evidence of endodontic disease | Procedure | Histological findings of dental pulp | Histological findings of dental hard tissues |
| --- | --- | --- | --- | --- | --- | --- | --- |
| #1 | LmandI1 | yes | AB | no | extraction | vital with coronal pulpitis | thickened dentin wall with partial pulp canal obliteration |
|  | LmandI2 | no | AB/discolored | Yes – wide pulp cavity, well-defined periapical lesion, inflammatory root resorption | extraction | pulp necrosis, neutrophilic and histiocytic inflammation | narrow dentin wall (arrested dentinogenesis) |
|  | LmandC | yes | AB | no | extraction | vital with coronal pulpitis | thickened dentin wall with partial pulp canal obliteration |
|  | LmandP1 | no | no | no | extraction | vital without inflammation | thickened dentin wall with partial pulp canal obliteration |
| #2 | RmandI2 | no | AB | no | extraction | unable to evaluate | thickened dentin wall with marked pulp canal obliteration |
|  | RmandC | yes | AB, draining tract | Yes - wide pulp cavity, well-defined periapical lesion and apical resorption | extraction | pulp necrosis | normal |
|  | RmandP1 | no | no | no | extraction | unable to evaluate | thickened dentin wall with marked pulp canal obliteration |

L – left, R – right, mand – mandibular, I – incisor tooth, C – canine tooth, P – premolar tooth
